# Elevated troponin I levels but not low grade chronic inflammation is associated with cardiac-specific mortality in stable hemodialysis patients

**DOI:** 10.1186/1471-2369-14-247

**Published:** 2013-11-09

**Authors:** Ahsan Alam, Andrea Palumbo, Istvan Mucsi, Paul E Barré, Allan D Sniderman

**Affiliations:** 1 Department of Medicine, Division of Nephrology, Royal Victoria Hospital, McGill University, 687 Pine Avenue West, Ross 2.39, Montreal, Quebec H3A 1A1, Canada; 2 Department of Medicine, Division of Cardiology, McGill University, Montreal, Quebec, Canada; 3 Institutes of Pathophysiology and Behavioral Sciences, Semmelweis University, Budapest, Hungary

## Abstract

**Background:**

Elevated cardiac troponin I (TnI) levels are associated with all-cause mortality in stable hemodialysis patients. Their relationship to cardiac-specific death has been inconsistent, and the reason for their elevation is not well understood. We hypothesized that elevated TnI levels in chronic stable hemodialysis patients more specifically track with cardiac mortality, and this mechanism is independent of other contributors of cardiac mortality, such as inflammation.

**Methods:**

We conducted a single-centre, cohort study of prevalent hemodialysis patients at a tertiary care hospital. Plasma TnI levels were measured with routine monthly blood tests in clinically stable patients for two consecutive months. Plasma TnI was measured by immunoassay and a value above the laboratory reference range (0.06 μg/L) was considered elevated. The primary outcome of death was adjudicated separately for this study, and classified as cardiac, non-cardiac, or unknown. Cox proportional hazard models were used to examine the association of TnI with the all-cause and cardiac-specific mortality, adjusting for potential confounders, including C-reactive protein (CRP) as a marker of inflammation.

**Results:**

Of 133 patients followed for a median of 1.7 years, there were 38 deaths (58% non-cardiac, 39% cardiac, 3% unknown). Elevated TnI was associated with adjusted HR for all-cause mortality of 2.57 (95% CI 1.30-5.09) and an adjusted HR for cardiac death of 3.14 (95% CI 1.07-9.2), after accounting for age, time on dialysis, diabetes status, prior coronary artery disease history, and C-reactive protein. Although CRP was also independently associated with all-cause mortality, it did not add prognostic information to TnI for cardiac-specific death.

**Conclusion:**

Elevated TnI levels are independently associated with cardiac and all-cause mortality in asymptomatic hemodialysis patients. The mechanism for this risk is likely independent of inflammation, but may reflect chronic subclinical myocardial injury or unmask those with subclinical atherosclerotic heart disease. Whether those with elevated TnI levels may benefit from additional investigations or more aggressive therapies to treat cardiovascular disease remains to be determined.

## Background

Cardiovascular disease (CVD) has been well recognized as the leading cause of mortality for patients on chronic hemodialysis, accounting for 45% of deaths [1-3]. Despite the burden of traditional CVD risk factors in this population, conventional (i.e. blood pressure or lipid lowering) and non-conventional treatment strategies (i.e. anemia and bone mineral disease management) have not been demonstrated to improve patient survival in this population [4]. Understanding the pathophysiologic mechanisms leading to this increased cardiac mortality need to be re-explored.

Subclinical myocardial ischemia and injury may be under-recognized in otherwise stable patients receiving renal replacement therapy. In hemodialysis, cardiac troponin T and troponin I (TnI) levels have been extensively studied. These studies are often heterogeneous, particularly with respect to TnI, and do not account for non-traditional cardiac risk factors, such as inflammation [5].

The aim of our study was to examine the association of elevated TnI levels in stable patients receiving maintenance hemodialysis therapy with cardiac-specific mortality, and discern if any increased risk was independent of inflammation status. Using a cohort study design, we measured plasma TnI levels with routine pre-dialysis monthly blood tests, including CRP, and tracked all patient outcomes, specifically adjudicating the cause of all deaths as cardiac or non-cardiac.

## Methods

### Study design

This was an observational, single-centre cohort study of prevalent hemodialysis patients at a tertiary care hospital. All clinically stable (i.e. non-hospitalized) chronic hemodialysis patients available both in July and August 2009 were followed prospectively until July 2012. This study was approved by the Research Ethics Board of the McGill University Health Centre. All blood tests were measured routinely in the hemodialysis unit at the time of the study and processed through the hospital’s central laboratory. Thus the institutional REB waived individual patient consent for use of existing clinical data. The study was conducted according to the Helsinki declaration for medical research in humans.

### Study participants

The inclusion criteria were all adult patients (age 18 years or older) receiving chronic hemodialysis irrespective of dialysis prescription, frequency, or vascular access. All patients in our dialysis unit received dialysis using a high flux membrane. Patients that were hospitalized or did not have a routine monthly blood test were excluded (N = 10).

### Data collection

Detailed data on demographic characteristics, medical history, medications, and routine laboratory tests from dialysis monthly blood work (complete blood count, serum electrolytes, iron profile, ferritin, albumin, calcium, phosphate, intact parathyroid hormone, and urea reduction ratio) were collected at baseline. If required, any missing data was captured from the hospital electronic medical record system or medical chart review. At the time of the study, C-reactive protein (CRP) was included as part of the routine dialysis monthly blood work on a bimonthly basis.

Plasma TnI levels were drawn pre-dialysis in clinically stable patients for two consecutive months. Patients with a hospital visit in the prior two weeks were not enrolled in this cohort. We reviewed all presentations to hospital in the prior two months. Sixteen patients had a troponin value measured in the prior two months, with only 5 patients showing any elevation, but all returned to within ‘normal-range’ by study initiation. In retrospect, one of the 5 individuals enrolled was found to have been admitted to the coronary care unit within the prior two months. This individual’s TnI normalized by study inclusion (TnI level <0.06) and he was included in the study analysis. Troponin I was measured by immunoassay (Beckman Coulter, Inc.) by the central laboratory in the hospital. Samples were assayed immediately, and were not frozen. The mean value from both months was used for the analysis. A value above the laboratory reference range (0.06 μg/L) was considered elevated.

### Outcome measures

The primary outcome for this study was cardiac-specific mortality. A secondary outcome was all-cause mortality. The date of all deaths is captured prospectively by the dialysis unit. The cause of death was ascertained by both dialysis and medical chart review by study investigators (AP, AA and AS) who were blinded to the TnI data. Adjudication as to the cause of death was by consensus and classified as ‘cardiac’ , ‘non-cardiac’ , or ‘unknown’.

Criteria for determining cardiac death required recognition of an acute coronary syndrome, congestive heart failure, or a fatal arrhythmia. For all in-hospital deaths, a review of all laboratory tests, including cardiac enzymes, as well as available electrocardiograms and cardiac imaging studies, such as coronary angiography, were used to establish a cause of death. For deaths outside the hospital we reviewed the last hemodialysis treatment run sheets and nursing or physician notes, or if available, ambulance and emergency room notes to identify any support for a cardiovascular death. Rarely, autopsy data was available to provide a definitive cause of death. The presence of an alternative cause of death or any withdrawal from dialysis was classified as non-cardiac.

### Statistical analysis

Baseline demographics and clinical characteristics are presented as means or medians and proportions, as appropriate. Natural log transformations of both TnI and CRP were performed due to their skewed distribution. Cox proportional hazard models were used for time-to-event analyses for both cardiac-specific and all-cause mortality outcomes. Proportional hazards assumptions were tested using log-negative-log survival plots. All models assessing the association of troponin I with mortality were adjusted for clinically relevant confounders and selected covariates that were significant from univariate analyses at a p-value less than 0.10. Censoring occurred for patients that transferred to another hemodialysis unit or switched dialysis modality (N = 6), had a kidney transplant (N = 5), or reached the end of the observation period (July 1, 2012).

Several sensitivity analyses were performed. The analyses were repeated using tertiles of TnI, a TnI cutoff of 0.10 μg/L, and natural log-transformed TnI as a continuous measure (Additional file 1: Table S1). Serum albumin was also examined as a marker of inflammation-malnutrition. All models were re-analyzed with serum albumin added to the adjusted model with CRP, and adjusting for serum albumin without CRP (Additional file 2: Table S2).

All analyses were done using SAS software version 9.2 (SAS Institute Inc. Cary, North Carolina).

## Results

In this stable hemodialysis population there were 36 patients (27%) with a TnI level greater than 0.06 μg/L. The mean TnI level was 0.06 ± 0.11, with a median value of 0.03 (IQR 0.02-0.06) mcg/L. Table 1 describes the demographic and baseline variables in those with and without an elevated TnI level. The prevalence of diabetes mellitus was significantly higher in those with a higher TnI level (58 vs. 37%, p = 0.03). The mean dialysis dose measured by urea reduction ratio was adequate in both groups, but was lower in those with an elevated TnI (70% vs. 74%, p = 0.03). C-reactive protein was significantly higher in those with a TnI level above the normal range (median 8.9 vs. 5.0, p = 0.03). Patient age, gender, a history of coronary or peripheral vascular disease, hypertension, dyslipidemia, smoking status, and time on dialysis did not associate with a higher TnI level. Although urea reduction ratio was lower in those with higher TnI, it was not associated with all-cause or cardiac-specific mortality in univariate analyses, and thus was not included as a confounder in multivariable models.

**Table 1:** Baseline patient study characteristics stratified by troponin I level elevation

	**TnI < 0.06**	**TnI ≥ 0.06**	**p**
	**N = 97**	**N = 36**	
Age, years	68.4 ± 14.0	65.4 ± 17.1	0.30
Male (%)	54 (56)	26 (72)	0.08
CAD history (%)	31 (32)	10 (28)	0.64
PVD history (%)	14 [16]	5 [18]	0.81
Diabetes mellitus (%)	36 (37)	21 (58)	0.03
Hypertension (%)	75 (86)	27 (93)	0.33
Dyslipidemia (%)	43 (49)	15 (51)	0.83
Smoking status (%)			0.15
Never	60 (71)	15 (52)	
Current	13 [15]	7 (24)	
Ex-Smoker	11 [13]	7 (24)	
Dialysis vintage, median years	2.0 (0.9-4.0)	2.9 (1.2-4.2)	0.57
Hemoglobin, g/L	111 ± 16	112 ± 19	0.81
Ferritin, μg/L	607 ± 425	505 ± 344	0.12
Albumin, g/L	32.1 ± 4.5	30.4 ± 5.3	0.08
Calcium total, mmol/L	2.19 ± 0.16	2.18 ± 0.09	0.81
Phosphorus, mmol/L	1.57 ± 0.48	1.54 ± 0.58	0.80
Urea reduction ratio, %	74.2 ± 8.0	70.2 ± 10.1	0.03
CRP, median mg/L	5.0 (1.7-13.4)	8.9 (4.1-21.3)	0.03

*CAD*, Coronary artery disease; *PVD*, Peripheral vascular disease; *CRP*, C-reactive protein.

Data are presented as frequency (proportion), means ± SD, or median (interquartile range).

The median observation period was 1.7 years (interquartile range 1.2 to 2.7 years). There were 38 deaths amongst 133 study participants. Of the 38 deaths, 22 patients were hospitalized at the time of death; the causes of death for 2 patients were confirmed by autopsy. Fifteen deaths were adjudicated as cardiac (N = 7) or sudden cardiac death (N = 8), and 22 were non-cardiac in nature. A cause could not be determined in one individual, and thus classified as non-cardiac for the purpose of our analysis.

A TnI level greater than 0.06 μg/L was associated with an increased hazard ratio (HR) of 2.57 (95% CI 1.30-5.09) for all-cause mortality even after adjusting for potential confounders, including CRP (Table 2). The association of TnI with cardiac-specific mortality was even stronger with an adjusted HR of 3.14 (95% CI 1.07-9.20). C-reactive protein was also independently associated with all-cause mortality, adjusted HR 1.37 (95% CI 1.07-1.75) per natural log increase. However, after adjusting for TnI level, CRP did not associate with cardiac-specific mortality. Diabetes mellitus was also independently associated with cardiac-specific mortality and trended towards a significant association with all-cause mortality. Variables such as gender, prevalent hypertension, dyslipidemia, or smoking status were not associated with mortality in univariate models, thus they were not considered further in our multivariable analysis.

**Table 2:** Cox proportional hazard ratios for the association of troponin I level with all-cause and cardiac-specific mortality

	**All-cause mortality**		**Cardiac-specific mortality**	
**Model**	**Unadjusted**	**Adjusted**	**Unadjusted**	**Adjusted**
	**HR (95% CI)**	**HR (95% CI)**	**HR (95% CI)**	**HR (95% CI)**
TnI ≥0.06	2.83 (1.49-5.37)*	2.57 (1.30-5.09)*	4.04 (1.46-11.2)*	3.14 (1.07-9.20)*
Age (per year)	1.01 (0.99-1.04)	1.01 (0.99-1.04)	1.00 (0.97-1.04)	1.00 (0.96-1.04)
Months on dialysis	0.99 (0.91-1.08)	1.01 (0.93-1.10)	1.01 (0.90-1.14)	1.07 (0.95-1.20)
CAD history	1.70 (0.90-3.23)†	1.60 (0.84-3.07)	1.85 (0.67-5.09)	1.71 (0.61-4.82)
Diabetes mellitus	2.14 (1.12-4.08)*	1.77 (0.89-3.51)†	4.40 (1.40-13.8)*	4.33 (1.22-15.3)*
CRP (per natural log)	1.40 (1.11-1.70)*	1.37 (1.07-1.75)*	1.27 (0.88-1.82)	1.20 (0.82-1.76)

*TnI*, Troponin I; *CAD*, Coronary artery disease; *CRP*, CRP.

Adjusted model includes all covariates listed in the table.

*p ≤ 0.05; †p ≤ 0.10.

**Figure 1:**
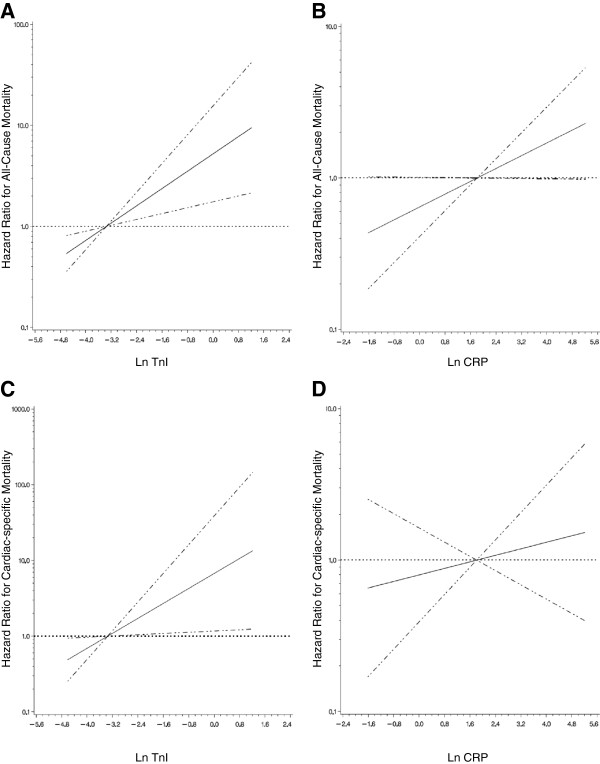
**The association of log-transformed troponin I and C-reactive protein with either all-cause or cardiac-specific mortality.** Estimated adjusted hazard ratio (solid line) with 95% confidence intervals (dashed lines) for the association of: **A)** natural log-transformed troponin I (LnTnI) with all-cause mortality; **B)** natural log-transformed C-reactive protein (LnCRP) with all-cause mortality; **C)** LnTnI with cardiac-specific mortality; **D)** LnCRP with cardiac-specific mortality. The median level of TnI or CRP was used as a reference point for the calculation of all hazard ratios. Non-linear relationships were first examined using restricted cubic splines (knots = 3, p < 0.001 for the test of linear relation). The models are adjusted for age, time on dialysis, coronary artery disease history, diabetes mellitus status, and either LnTnI or LnCRP, as appropriate.

Sensitivity analyses were conducted to examine the robustness of the relationship between TnI and patient outcomes. Examining TnI as a continuous (natural log-transformed) variable showed similar results (Figure 1), as did the cutoff of 0.10 μg/L (Additional file 1: Table S1). Tertiles of TnI showed a dose–response with both all-cause and cardiac-specific mortality, but statistical significance was not met, likely due to a loss of power from our limited sample size. Serum albumin (per 1 g/L) was also inversely associated with all-cause mortality, HR 0.90 (95% CI 0.85-0.95), but not cardiac-specific mortality, HR 0.94 (95% CI 0.84-1.04). When serum albumin was added to a fully adjusted model for all-cause mortality, it remained significant, but did not confound the association of TnI with all-cause mortality. The significance of CRP with all-cause mortality was lost when serum albumin was added to the model (Additional file 2: Table S2).

## Discussion

In this study, we report a strong association between elevated TnI level and all-cause and cardiac-specific mortality in non-hospitalized and clinically stable patients receiving maintenance hemodialysis. This relationship was independent of other potential confounders, including inflammation measured by either CRP or serum albumin. It is notable that although CRP did associate with all-cause mortality it did not show an independent association with cardiac-specific mortality after adjusting for TnI.

Cardiac troponins are a mainstay in detecting acute cardiac injury in those with and without kidney disease. In otherwise stable hemodialysis patients elevated troponin levels have also been shown to predict adverse outcomes, even in the absence of evident cardiac ischemia. The prevalence, threshold and significance of TnI elevation remain uncertain.

There have been numerous studies that have attempted to examine this issue, even prompting a meta-analysis on cardiac troponins in patients with end-stage renal disease. [5] This analysis highlighted that the majority of studies focused on troponin T, which is indeed associated with total and cardiac mortality. Troponin T is elevated in patients with impaired renal clearance and on hemodialysis, requiring the normal cutoff to be different than the general population. Fewer studies have examined TnI. Many of these TnI studies are limited by small sample size, low event rates, or short observation period [6-14]. Some applied a much higher TnI cutoff (0.10-2.8 ng/mL) often due to the use of older assays [15,16]. The meta-analysis highlighted the heterogeneity between studies, and particularly the estimates of cardiac-specific death risk being very highly variable. None of these studies earlier took into account CRP or inflammation status.

Boulier et al. specifically examined the role of TnI enhancing the prognostic value of CRP [17]. In this study of 191 French hemodialysis patients, TnI was similarly associated with all-cause as well as cardiovascular deaths, although the method of adjudicating cause of death was not specified. Applying a TnI cutoff of 0.03 μg/L, they found an association with death, with the greatest risk in those also having a CRP level above 10 mg/L. Our analysis extends their findings, also demonstrating that the risk associated with TnI is linear across its range, but most importantly showing that this risk is entirely independent of CRP. This would suggest that attention on inflammation, as measured by CRP, in trying to explain cardiovascular risk in patients on hemodialysis may not warrant the most focus.

As expected, diabetes mellitus status was also strongly and independently associated with cardiac-specific mortality, and more so than with all-cause mortality. Although inflammation may be highly prevalent in those with diabetes, our analysis suggests that these parameters are independent in their association with mortality. Interestingly, patient age did not associate with mortality in our cohort. Patients who died within the observation period were indeed older (mean age 70.4 ± 15.1 vs. 66.5 ±14.8, p = 0.17) than those who remained alive, but this did not reach statistical significance in survival analysis models. The lack of association may in part be related to our exclusion of hospitalized/unstable patients, which we suspect excluded older patients. Also, the age of our dialysis cohort ranged from 26–93 years of age. These extremes of age may represent a lower risk population with respect to cardiac disease, due to selection and survival bias. Similarly, a history of coronary artery disease (CAD) did not predict cardiac mortality. We characterized CAD from chart review, but it is unclear whether CAD documentation by physicians was complete and comprehensive. Furthermore, severity and treatment of CAD was not captured, and this may be a greater and more relevant influence on outcomes.

The causes of troponin release in the absence of myocardial necrosis are numerous (e.g. congestive heart failure, pericarditis, tachyarrhythmia, pulmonary embolism, sepsis, etc.), and likely results from increased myocyte membrane permeability. Given that TnI levels remained elevated over at least two months in our patients, we would expect these traditional causes to become evident or to be associated with clinical symptoms. Instead, we suggest that repeated and subclinical ischemia-reperfusion injury from the hemodynamic effects of intermittent hemodialysis, particularly in those with a vulnerable myocardium or occult atherosclerotic disease, to be a more rational hypothesis. Dialysis-specific factors may be related to intradialytic hyper- or hypotension, aggressive ultrafiltration, electrolyte shifts, or even bioincompatibility; however, our study was not designed to address this question. The hypothesis of “uremic cardiopathy” is also not clearly supported by our data as the dialysis adequacy would be considered adequate for both TnI groups. The issue of dialysis-induced myocardial stunning has also been linked to elevated troponin T levels, but its association with long-term CVD or mortality outcomes remains to be evaluated [18].

There are several strengths of this study. We characterized TnI levels in a stable hemodialysis population with close longitudinal follow up. Troponin I levels were measured on two consecutive monthly visits to establish a stable baseline level. C-reactive protein was also measured concurrently as a marker of inflammation, and our analyses were repeated with serum albumin as well. The cause of death was classified as cardiac or non-cardiac on the basis of individual patient charts reviews, with the adjudicators blinded to the TnI level.

There are limitations to the study as presented. This cohort is drawn from a single-centre, and may not be generalizable to all dialysis populations. Although our results are robust, both in primary and secondary analyses, we did have limited power to explore subgroups or additional potential confounders. It is unclear whether an elevated TnI is determined by patient-specific factors, such as comorbidities, or whether the hemodialysis treatment or complications related to treatment (e.g. intradialytic hypotension) is the primary contributor to cardiac risk. We did not capture hemodynamic factors during hemodialysis sessions, or repeat TnI levels before and after dialysis. Although we did repeat TnI levels over two months, serial measurements over longer time were not captured, and may allow for more accurate detection of myocardial injury. In our institution CK-MB is not routinely measured for the diagnosis of an acute coronary syndrome, and we did not perform other diagnostic or invasive cardiac testing. Our study population was selected if they had no presentation to our hospital in the prior two weeks; however, we could not ascertain if they presented to another institution or had a myocardial event that was not investigated in the preceding two months. Also, we did not capture non-fatal outcomes, as we felt this would be difficult to adjudicate accurately with respect to cardiac and non-cardiac in nature.

## Conclusions

Given the magnitude and burden of CVD in patients with ESRD, it is essential to identify reliable measures of increased risk. Elevated TnI levels provide independent prediction of cardiac risk. More important, elucidating the underlying pathophysiologic processes that lead to TnI elevation is critical. Our observational study cannot directly justify a change in clinical practice; however, this study adds evidence to support a more comprehensive approach to cardiac risk stratification in dialysis patients, which may include such tests as exercise or pharmacologic stress testing. We postulate that ischemia-reperfusion injury should also be a focus of further investigation in these otherwise stable chronic hemodialysis patients. The implication of these results would allow for interventional studies to address potentially modifiable factors, such as dialysis prescription or modality changes, indications for targeted risk stratification, such as functional cardiac testing, or more aggressive medical management in this vulnerable population.

## Competing interest

None of the authors have any financial competing interests in relation to the publication of this study. AA was supported from institutional funds from the Research Institute of the McGill University Health Centre. None of the authors receive any funding, honoraria, or hold any stocks, shares, or patents that may be impacted by the publication of this manuscript.

The results presented in this paper have not been published previously in whole or part, except in abstract format.

## Authors’ contributions

PB, AS and AA designed the study. AP, AA and AS were involved in the acquisition of data, statistical analysis, and interpretation of the results. All authors participated in drafting the manuscript or revising it critically for important intellectual content, and all authors approved the final manuscript.

## Pre-publication history

The pre-publication history for this paper can be accessed here:

http://www.biomedcentral.com/1471-2369/14/247/prepub

## Supplementary Material

Additional file 1: Table S1 Cox proportional model hazard ratios (95% confidence intervals) for varying troponin I transformations for both all-cause and cardiac-specific mortality. Click here for file

Additional file 2: Table S2 Cox proportional hazard models for the association of troponin I level with all-cause and cardiac-specific mortality, adjusting for C-reactive protein and albumin. Click here for file
