# Supplementary material for: Elevated troponin I levels but not low grade chronic inflammation is associated with cardiac-specific mortality in stable hemodialysis patients
Source: BMC Nephrol. 2013 Nov 9;14:247. doi: 10.1186/1471-2369-14-247 (PMC4226253; doi:10.1186/1471-2369-14-247)
Supplement: Additional file 1: Table S1 — Cox proportional model hazard ratios (95% confidence intervals) for varying troponin I transformations for both all-cause and cardiac-specific mortality. [file 1471-2369-14-247-S1.doc]

Additional file 1: Table S1. Cox proportional model hazard ratios (95% confidence intervals) for varying troponin I transformations for both all-cause and cardiac-specific mortality

|  | **Unadjusted** | **Model 1** | **Model 2** |
| --- | --- | --- | --- |
| **All-cause Mortality** | | | |
| Mean TnI (natural log-transformed) | **1.80 (1.37-2.37)** | **1.88 (1.41-2.49)** | **1.64 (1.18-2.27)** |
| TnI Tertiles  mid vs. low  high vs. low | 2.32 (0.85-6.39)  **4.05 (1.50-10.9)** | 2.22 (0.79-6.21)  **3.99 (1.48-10.8)** | 1.60 (0.56-4.58)  2.54 (0.91-7.14) |
| TnI cutoff  ≥0.06  ≥0.10 | **2.83 (1.49-5.37)**  **3.99 (1.98-8.06)** | **2.98 (1.57-5.67)**  **4.38 (2.15-8.92)** | **2.44 (1.25-4.77)**  **3.23 (1.54-6.77)** |
| **Cardiac-specific Mortality** | | | |
| Mean TnI (natural log-transformed) | **1.96 (1.29-3.00)** | **2.09 (1.32-3.31)** | **1.77 (1.05-2.98)** |
| TnI Tertiles  mid vs. low  high vs. low | 1.94 (0.38-9.99)  4.58 (0.97-21.6) | 1.94 (0.37-10.3)  4.57 (0.96-21.7) | 1.38 (0.24-7.93)  2.72 (0.52-14.3) |
| TnI cutoff  ≥0.06  ≥0.10 | **4.04 (1.46-11.2)**  **4.88 (1.66-14.3)** | **4.09 (1.47-11.3)**  **5.07 (1.69-15.2)** | **3.14 (1.07-9.20)**  **3.07 (1.00-9.43)** |

**Model 1**: Adjusted for age, months on dialysis
**Model 2**: Adjusted for variables in model 1 + coronary artery disease history, diabetes mellitus, C-reactive protein (natural-log transformed)
