# Supplementary material for: Elevated troponin I levels but not low grade chronic inflammation is associated with cardiac-specific mortality in stable hemodialysis patients
Source: BMC Nephrol. 2013 Nov 9;14:247. doi: 10.1186/1471-2369-14-247 (PMC4226253; doi:10.1186/1471-2369-14-247)
Supplement: Additional file 2: Table S2 — Cox proportional hazard models for the association of troponin I level with all-cause and cardiac-specific mortality, adjusting for C-reactive protein and albumin. [file 1471-2369-14-247-S2.doc]

Additional file 2: Table S2. Cox proportional hazard models for the association of troponin I level with all-cause and cardiac-specific mortality, adjusting for C-reactive protein and albumin

|  | **All-cause mortality** | | **Cardiac-specific mortality** | |
| --- | --- | --- | --- | --- |
| **Model** | **Model 1 HR (95%CI)** | **Model 2**  **HR (95% CI)** | **Model 1 HR (95%CI)** | **Model 2**  **HR (95% CI)** |
| TnI ≥0.06 | 2.91 (1.48-5.69)* | 2.82 (1.41-5.61)* | 4.09 (1.34-12.47)* | 4.20 (1.32-13.4)* |
| Age (per year) | 1.02 (0.99-1.04) | 1.02 (0.99-1.04) | 1.01 (0.97-1.05) | 1.01 (0.97-1.05) |
| Months on dialysis | 1.02 (0.94-1.10) | 1.02 (0.94-1.10) | 1.09 (0.96-1.23) | 1.09 (0.96-1.23) |
| CAD history | 1.56 (0.81-3.02) | 1.56 (0.81-3.03) | 1.85 (0.63-5.43) | 1.83 (0.62-5.40) |
| Diabetes mellitus | 1.73 (0.86-3.49) | 1.74 (0.86-3.50) | 6.10 (1.37-27.1)* | 6.08 (1.37-27.1)* |
| CRP (per natural log) | - | 1.06 (0.79-1.41) | - | 0.96 (0.59-1.56) |
| Serum albumin (per 1 g/L) | 0.90 (0.84-0.96)* | 0.90 (0.83-0.98)* | 0.95 (0.83-1.07) | 0.94 (0.80-1.10) |

TnI: troponin I; CAD: coronary artery disease; CRP: C-reactive protein
Adjusted models includes all covariates listed in the table.
*p≤0.05
